# Clinical Features and Correlates of Outcomes for High-Risk, Marginalized Mothers and Newborn Infants Engaged with a Specialist Perinatal and Family Drug Health Service

**DOI:** 10.1155/2012/867265

**Published:** 2012-11-22

**Authors:** Lee Taylor, Delyse Hutchinson, Ron Rapee, Lucy Burns, Christine Stephens, Paul S. Haber

**Affiliations:** ^1^School of Psychology, Macquarie University, Sydney, NSW 2113, Australia; ^2^National Drug and Alcohol Research Centre, Faculty of Medicine, University of New South Wales, Sydney, NSW 2052, Australia; ^3^Drug Health Services, Royal Prince Alfred Hospital, Camperdown, NSW 2050, Australia; ^4^Sydney Medical School, Camperdown, NSW 2050, Australia

## Abstract

*Background*. There is a paucity of research in Australia on the characteristics of women in treatment for illicit substance use in pregnancy and the health outcomes of their neonates. *Aims*. To determine the clinical features and outcomes of high-risk, marginalized women seeking treatment for illicit substance use in pregnancy and their neonates. *Methods*. 139 women with a history of substance abuse/dependence engaged with a perinatal drug health service in Sydney, Australia. Maternal (demographic, drug use, psychological, physical, obstetric, and antenatal care) and neonatal characteristics (delivery, early health outcomes) were examined. *Results*. Compared to national figures, pregnant women attending a specialist perinatal and family drug health service were more likely to report being Australian born, Aboriginal or Torres Strait Islander, younger, unemployed, and multiparous. Opiates were the primary drug of concern (81.3%). Pregnancy complications were common (61.9%). Neonates were more likely to be preterm, have low birth weight, and be admitted to special care nursery. NAS was the most prevalent birth complication (69.8%) and almost half required pharmacotherapy. *Conclusion*. Mother-infant dyads affected by substance use in pregnancy are at significant risk. There is a need to review clinical models of care and examine the longer-term impacts on infant development.

## 1. Introduction

Illicit drug use is a major problem in young adults and increasingly in young women [1]. It is well documented that the myriad of health problems associated with illicit drug use is of serious concern, especially during the antenatal and postnatal period. Unfortunately, the number of pregnancies that are affected by substance use appears to be considerable [2, 3]. A survey of maternity facilities in New South Wales and the Australian Capital Territory in 2004 [4] identified that up to 1.3% of pregnancies were affected by perinatal substance dependence. The 2004 National Household Drug Survey found that 6% of women had used some form of illicit drug whilst pregnant or breastfeeding within the previous 12 months [1]. 

Perinatal substance use has been found to be associated with a higher incidence of pregnancy complications, including miscarriage, stillbirth, fetal growth retardation, decreased birth weight and head circumference, premature delivery, meconium staining, cerebral infarctions, maternal health problems, and maternal/neonatal infections and neonatal mortality [5–10]. Neonates born to mothers affected by substance use problems are more likely to be admitted to special care nurseries, have longer stays in hospital, and are at increased risk of neonatal abstinence syndrome (NAS) compared to nonexposed neonates [11–14]. In addition to the adverse short-term outcomes, it has also been suggested that in the longer term, children and young people exposed to illicit drugs in utero have poorer neurodevelopmental outcomes, including health problems, behavioural and emotional problems, attention deficits, learning difficulties, and language and speech difficulties [15–23]. Whether the cause of these outcomes is the perinatal substance use itself or the aftermath of factors typically associated with illicit drug use, such as economic and social disadvantage, polydrug use, chaotic lifestyle, poor antenatal health, nutrition and health care, low social support, biological factors and parenting deficits, remains unclear [18, 24, 25].

It is well known that women who use illicit substances during the prenatal period engage late with antenatal care providers, attend antenatal visits infrequently, and in some cases, receive no antenatal care, first presenting to hospital in labour [26–31]. Factors commonly associated with the illicit substance use lifestyle, such as major life stressors, poor coping skills, limited social support systems, frequent changes in residence/employment, unemployment, long standing substance abuse and polydrug use, domestic violence, easy access to illicit substances, and identity/self esteem issues [32] are all probable reasons as to why these women do not access specialist services earlier. It is well documented in the literature that little or no prenatal care is associated with poor pregnancy outcomes [1, 30]. 

Prenatal substance abuse and dependence have been associated with an increased risk for child maltreatment/abuse and involvement with child protection services [33–35]. Risk of child protection is often exacerbated by domestic violence and poor maternal mental health, which are also commonly associated with maternal substance use [36, 37]. A recent Australian study investigating the risk of child protection involvement for children born with NAS and its interrelationship with maternal mental health and exposure to violence found that infants who received a diagnosis of NAS were at greater risk of having received a substantiated child maltreatment allegation and entering foster care [3]. 

There is a paucity of research in Australia which details the demographic and drug use characteristics, obstetric history, and maternal and neonatal outcomes of women with substance use problems in pregnancy. This study, therefore, aims to document the demographic, drug use characteristics, antenatal care and delivery outcomes of a high risk, marginalized subset women affected by illicit substance use and abuse in pregnancy, and where data are available, to compare those characteristics with the general population in Australia. The study also aims to document the short-term birth outcomes of neonates born to this subset of substance using pregnant women and to compare these outcomes with general Australian population data. Understanding more about these high-risk, marginalized substance-affected mother-infant dyads will help to better direct treatment and intervention services and potentially lessen the burden on the health system.

## 2. Materials and Methods

### 2.1. Study Design and Data Source

This is a prospective study examining the characteristics of a high-risk, marginalized subset of pregnant women with a known history of substance abuse and dependence (substance-exposed (SE) women) who accessed antenatal care from a specialist perinatal drug health service within a large public hospital in Sydney, Australia. All women attending this hospital for obstetric care with alcohol and/or drug dependence (except for isolated nicotine dependence) are routinely referred to this service. The specialist clinic runs in conjunction with the general antenatal clinic and offers women individualised treatment plans that address substance use and associated psychosocial issues. All referrals are assessed by a Clinical Nurse Specialist and a multidisciplinary case management plan is developed. There are regular case management meetings attended by midwives, neonatologists, social workers, and addiction medicine clinicians during pregnancy and the early postnatal period. A team of specialist consultants within the Perinatal and Family Drug Health Clinic developed a systematic data collection process with the purpose of evaluating clinical interventions and services provided to women, to monitor clinical outcomes and direct service improvements. The Perinatal and Family Drug Health Data Form records information on the mother and her pregnancy, overall antenatal care, birth and delivery, the postnatal period, and neonatal variables. Data were collected by a Drug Health Perinatal Clinical Nurse Consultant in three sections: (1) initial assessment (demographic, psychosocial issues, pregnancy details, substance use, and pharmacotherapies); (2) antenatal (pregnancy care, services involved, and complications); (3) postnatal (birth and neonatal outcomes). Women who attended the clinic (*n* = 139) and gave birth between February 2004 and September 2007 were included in the analyses.

### 2.2. Maternal Variables

Characteristics of the mother included: age, country of birth, indigenous status, employment, relationship status, parity and care arrangements, previous child protection involvement, substance use history and treatment, presence of communicable disease, medical complications and other psychosocial issues. 

### 2.3. Antenatal Care and Delivery Variables

Characteristics of antenatal care included gestation at first visit, number of antenatal visits, and complications during pregnancy. Characteristics of the delivery included whether the delivery was booked, whether labour was spontaneous or induced, type of delivery, and pain relief.

### 2.4. Neonatal Variables

Characteristics of the neonate included gestational age, birth weight, Apgar score at one and five minutes, delivery complications including whether resuscitation was required, transfers to Special Care Nursery, highest NAS score and whether treatment was required, length of stay in hospital, feeding regime, and the discharge care arrangements of the neonate. 

For the purposes of this study, the de-identified data forms of 139 women and their neonates were examined. Approval for the study was received from Macquarie University Ethics Review Committee (Human Research); the University of New South Wales Human Research Ethics Committee and the Ethics Review Committee (RPAH Zone) of the Sydney South West Area Health Service. 

## 3. Results

Where data were available, the results were compared with figures published in *Australia's mothers and babies (AMAB) 2007* [38]. *AMAB 2007* is the 17th annual report on pregnancy and childbirth in Australia providing national information on women who gave birth and the characteristics and outcomes of their babies. In 2007, 289,496 women gave birth to 294,205 babies in Australia. The study results were also compared to NSW data in *AMAB 2007* to ascertain the presence of possible state-specific differences. Given no major differences were detected, national data were used as the main point of comparison. Statistical analyses were performed using SPSS. *T*-tests and chi-square analyses were used to determine whether there were differences between SE women and national population data. The national data set includes sample sizes, percentages, and means where appropriate. Standard deviations were not published and therefore, it was not possible to calculate 95% confidence intervals.

### 3.1. Maternal Characteristics

Compared to the national sample, SE women were significantly younger at delivery (range 16–45 years; *p* < 0.05), were more likely to be Australian-born (*p* < 0.001), and more likely to identify as Aboriginal and Torres Strait Islander (*p* < 0.001; Table 1). Most SE women (90.6%) were unemployed. Mental health issues were reported by 38 (27.3%) women, domestic violence by 19 (13.7%), and 11 (7.9%) were homeless. Over half (62.6%) of the SE women were multiparous and were likely to have higher parity than women in the national sample (*p* < 0.05). Approximately one-third of women (35.3%) reported past child protection involvement with other children and over half (58.9%) had at least one previous child in kinship or foster care. 

Opiates were the primary presenting drug problem for 81.3% of women; 8.6% had a primary cannabis problem; 5% had a primary alcohol problem (Table 2). The majority (84.8%) of women smoked throughout pregnancy (rates were significantly higher (*p* < 0.001) than in the national sample 16.6%); just under half (45%) used cannabis and a third (37.4%) used heroin. Most (91.4%) SE women reported polydrug use (use of two or more substances). 115 women (82.7%) received pharmacotherapy treatment for their substance use, of which 72.7% were enrolled in a methadone program and 8.6% were on buprenorphine. 

Hepatitis C antibodies were present in 83 (59.7%) women. Hepatitis C PCR was detected in almost half (49.6%) of the women. Hepatitis B surface antigen was detected in 2 (1.4%) women and 1 woman (0.7%) evidenced syphilis antibodies.

### 3.2. Antenatal Care and Delivery Variables

Women received the first occasion of antenatal care at an average gestation of 19.83 weeks and attended, on average, 6.5 antenatal visits (Table 3). Compared to the national sample, SE women were significantly less likely to have had five or more antenatal follow-up visits (*p* < 0.001) and more likely to have had no antenatal care at all (*p* < 0.001). A large proportion of SE women (61.9%) experienced complications in pregnancy. The most common complication was fetal distress (14.4%), followed by premature rupture of the membranes (11.5%) and intrauterine growth restriction (10.1%). Prenatal child at risk notifications were made in 41 cases (29.5%).

Most women (93.5%) had their delivery booked at hospital and statistics on the onset, type of labour, pain relief for labour, and operative delivery were similar to national data (Table 4). Most SE neonates were delivered vaginally, however, there was a higher proportion of breech presentations compared to the national sample (*p* < 0.05). The median maternal postnatal hospitalization for SE woman was 7.0 days (interquartile range: 6.0–8.0), which was more than two times as long a median stay than national samples values (*p* < 0.001). At least in part, this increased length of stay reflects NSW health policy to extend hospitalisation allowing for engagement of infant and maternal health care services.

### 3.3. Neonatal Variables

SE neonates were significantly more likely to have low birth weight (*p* < 0.001), be pre-term (*p* < 0.001), have an Apgar score of less than seven five minutes after birth (*p* < 0.001), and to have been admitted to a high care unit (Neonatal Intensive Care Unit or special care nursery) (*p* < 0.001; Table 5) compared with neonates in the national sample. Birth complications were common (78.4%); NAS being the most prevalent (69.8%). Most (89.7%) of the neonates required admission to a high care unit and almost half (48.9%) required pharmacotherapy. Ten percent of the neonates required special care for non-NAS-related complications. Almost half (48.9%) of the sample were breastfeeding at birth. 

The median length of stay in hospital for neonates was 9.0 days (interquartile range: 7.0–16.0), which was significantly longer than the length of stay of neonates in the national sample (*p* < 0.001; Table 6). Neonates with a short hospital stay (1–6 days) were significantly less likely to have received NAS pharmacotherapy (*p* < 0.001); neonates who received NAS pharmacotherapy were 6.2 times (CI: 2.0–19.5) more likely to have had a longer hospitalisation of 14–20 days (*p* < 0.001). There were no significant differences regarding NAS treatment for a hospital stay of 7–13 days. Whilst most (77.2%) neonates were discharged home into the care of their mother/both parents, the proportion was significantly lower than the national sample (*p* < 0.001). Three neonates from the study sample (2.2%) were transferred to another hospital, three died before discharge, three were discharged to a residential care unit with their mother, and 19 (13.7%) were assumed into care at or before discharge. Of those assumed into care, four (2.9%) went into kinship care and 15 (10.8%) into foster care. 13 (68.4%) of the 19 neonates assumed into care had at least one older sibling in care; five (26.3%) did not have any older siblings, and one (5.26%) had an older sibling in the care of his/her parents. The mothers and their newborns were linked in with a range of services at the time of discharge. Most common among these were drug and alcohol services, the NAS clinic and their local early childhood centre. 

## 4. Discussion and Conclusion

This study highlights the wide ranging psychosocial, obstetric and neonatal concerns which affect a high-risk, marginalized cohort of pregnant substance using women attending a specialist antenatal clinic of a large public hospital in Sydney, Australia. This is one of few Australian studies to document the characteristics of a high-risk subset of substance using women during pregnancy and the neonatal period. Unsurprisingly, the majority of SE women were from socially disadvantaged backgrounds, compared to national data presented in Australia's Mothers and Babies 2007 [38]. Specifically, they were more likely to be younger at the time of delivery, unemployed, Australian-born, indigenous, have higher parity, and report significant psychosocial issues and greater incidence of previous and current involvement with child protection services. These findings are consistent with the small body of existing literature published on Australian data [10–12].

 Determining the impact of specific classes of substance use in pregnancy is complicated as polydrug use is common [39, 40]. The majority of women in this sample presented with at least two substances of concern at the time of service engagement and well over half presented with three or more. Opiates were the principle drug of concern, which is in accordance with previous results from a large record linkage study examining illicit drug use in pregnancy in NSW, Australia [12]. The most common substances used consistently throughout pregnancy were nicotine, methadone, cannabis, heroin, and benzodiazepines. Of those using heroin, amphetamines and/or cocaine, most injected the drugs intravenously, suggesting a high frequency of use and increased risk of psychiatric comorbidity, poorer physical health, risky injection and unsafe sexual practices, HIV, criminal activity, and violence [41–44]. Almost all of the women in the cohort smoked cigarettes throughout their pregnancy, which is common in substance using populations and associated with an increased risk for other substance use [45]. This is also important with regard to improving screening and early detection of substance use during the perinatal period, as nicotine use would potentially be a marker for a more thorough inquiry about possible use of other substances.

In the general population, confirmation of pregnancy, whether planned or unplanned, typically precipitates a range of positive and proactive health and lifestyle changes, including altering patterns of substance use. Commonly women choose to either abstain or markedly reduce their intake of substances during pregnancy. The finding that continued use of a range of substances over the course of pregnancy was common, despite most receiving some form of pharmacotherapy for substance abuse and dependence, exemplifies the chronic and complex nature of substance use in this high-risk, marginalized subset of women. In turn, these results highlight the importance of raising the issue of pregnancy among high-risk populations of substance abusing women.

Most women in the study sample reported at least one antenatal visit and over half reported five or more visits. Whilst the level of prenatal care received was significantly less than that reported in the national sample, it is noteworthy that only a small percentage of women received no prenatal care at all. This is an important positive trend given that engaging women in prenatal care has the potential to reduce adverse outcomes commonly associated with perinatal substance use [2, 46–48].

Whilst pregnancy complications were high, there were no significant differences between the SE women and the national sample on the onset and type of labour, method of birth or whether analgesia was administered to relieve pain for labour. However, there were a significantly higher proportion of women with a breech presentation, which is consistent with findings from previous research [12]. The median duration of maternal postnatal hospital stay in our cohort was more than double that reported in the national sample, which is almost certainly a direct result of the higher incidence of maternal health concerns and neonatal complications. 

Neonatal complications were more common in the study sample when compared to national population data. The overrepresentation of prematurity (23.7% versus 8.1%), low birth weight (25.2% versus 7.9%), Apgar scores below seven at five minutes (5.8% versus 1.4%) and higher admission rates to intensive or special care nursery (69.1% versus 14.5%), is not surprising, given that these perinatal complications have been associated with substance abuse and dependence in high-risk populations in previous research [5–8]. In addition, most of the neonates in the cohort were diagnosed with NAS. Again, this is not surprising given that opioids were the primary drug of concern at initial presentation and most women were receiving opioid treatment in pregnancy. It is well documented that the risk for NAS is greatest in infants exposed in utero to opioids such as heroin and methadone. Accordingly, recent research has documented a marked increase in the birth prevalence of NAS at a total population level over the past 25 years in Australia [3]. 

Neonates in this high-risk subset generally require significantly longer hospital stays than nonsubstance exposed neonates [49, 50]. This was the pattern identified for the neonates in the present cohort, with shorter stays (1–6 days) being associated with not receiving NAS pharmacotherapy. A mandatory hospital stay of five days was imposed for mother and neonate when the mother's substance use included benzodiezapines or opioids. Almost half of the sample required a longer stay of 7–13 days compared to the national sample. Interestingly, this was not specifically related to NAS pharmacotherapy and is likely to be due to the range of other non-NAS complications associated with perinatal substance use in this population subset. Notwithstanding, neonates requiring an extended stay of 14–20 days were 6.2 times more likely to have been receiving NAS treatment. Neonates who stayed longer than 28 days were those who had serious medical complications.These results appear consistent with the literature indicating that high-need neonates require greater resources [3, 9, 11]. Prolonged hospital stays place an additional burden on hospital resources and cause disruption to the neonate and family system.

Child protection concerns were high, evidenced by the fact that child at risk notifications were made during pregnancy or postnatally for almost half of mother-neonate dyads. Of the 19 neonates removed from maternal care, at birth or prior to hospital discharge, 18 exhibited NAS, supporting recent Australian findings that infants with NAS are at considerable risk for entering foster care [3]. Fourteen of the neonates that were placed outside the mother's care had existing siblings, with all but one of these neonates having at least one sibling already placed in foster or kinship care or adoption. This is consistent with previous research [51] and highlights the serious risk of child protection issues within this high-risk, marginalized subset of women affected by substance abuse/dependence during pregnancy.

There are several limitations of the current study. The pattern of substance use exposure was ascertained by maternal self-report and may have resulted in an underestimate of levels of use and abuse. Studies have demonstrated a difference between the accuracy of reporting on substance use during pregnancy and retrospectively after pregnancy has ended [52] and other studies comparing urine toxicology and self-report measures have shown that women are likely to under-report their usage of illicit substances [45]. Underrecognition of substance use may have led to underreferral so the findings are likely to be biased towards those with more severe and chronic problems, in particular, pregnant women using and/or being prescribed opiates, who were more easily detected by antenatal staff. Given the study involved a subset of high-risk, marginalized pregnant women attending a specialist substance use antenatal clinic in a large metropolitan city, it is not advisable to generalize the results to all pregnant, substance using women, and especially those with more moderate (or less) substance use abuse. Data were prospectively collected by clinicians using structured proforma, but some data were missing requiring retrospective review of medical records. The findings are therefore dependent upon thoroughness of the initial assessment, the standard documentation in patient case notes, and the precision during data entry from case notes. We were also unable to document posthospital fostering rates. 

In conclusion, the findings highlight that this marginalized subset of mother-infant dyads affected by substance use are extremely vulnerable and are at significant risk of a multitude of medical, social, and psychological issues. Although the women in this cohort were linked in with specialist services post discharge, they remained highly disadvantaged, with outcomes that were still well below the average obstetric population. The short-term outcomes found in this study are consistent with previously published Australian studies. Taken together, there remains a need to identify strategies for more successful intervention with this high-risk population and to conduct research to document its effects. Continued monitoring of drug use patterns and their effects in the antenatal population appears warranted. Finally, further research is needed to document the longer term developmental outcomes of these high-risk infants, including the interplay of parenting stress and parental mental health.

## Figures and Tables

**Table 1:** Demographic and psychosocial characteristics.

Characteristics	Result	National data
Maternal age at delivery, years	28.7	29.9*
% ATSI^†^	40 (28.8)	3.8**
% Australian-born	85.6	75.2**
% Currently in relationship	72 (51.8)	—
% Unemployed	111 (90.6)	—
% Reporting mental health issues	38 (27.3)	—
% Reporting domestic violence	19 (13.7)	—
% Currently homeless	11 (7.9)	—
% Reporting previous DoCS^††^ involvement	49 (35.3)	—
% Have previous child in kinship/foster care	58.9	—
Parity		
None	37.4	41.6*
One	30.9	33.5*
Two	17.3	15.4*
Three	4.3	5.5*
Four or more	10.1	3.8*

Study data presented as number (%); data from national data presented as % only unless otherwise stated.

*p value < 0.05; **p value < 0.001.

^†^ATSI: Aboriginal and Torres Strait Islander.

^††^DoCS: Department of Community Services.

**Table 2:** Substance use and treatment during pregnancy.

Characteristics	Result	National data
Primary drug at initial presentation		
% Methadone	43.2	—
% Heroin	33.1	—
% Cannabis	8.6	—
% Buprenorphine	5.0	—
% Alcohol	3.6	—
Method of use for primary drug		
% IVDU^†^	34.5	—
Secondary drug at presentation		
% A second opiate	20.8	—
% Benzodiazepines	15.1	—
% Cannabis	15.1	—
% Cocaine	5.8	—
Substances used during pregnancy		
% Nicotine	84.9	16.6**
% Methadone	74.8	—
% Marijuana	45.0	—
% Heroin	37.4	—
% Benzodiazepines	20.1	—
% Amphetamines	12.9	—
% Alcohol	12.9	—
% Cocaine	11.5	—
% Buprenorphine	10.8	—
% Ecstasy	1.4	—
% Inhalants	0	—
% Polydrug use	91.4	—
Pharmacotherapy for substance use		
% Methadone	72.7	—
% Buprenorphine	8.6	—
% Naltrexone/Benzo	1.4	—
% Nil treatment	17.3	—
Communicable disease		
% Any type	62.6	—
% Hepatitis C antibodies	59.7	—
% Hepatitis B surface antigen	1.4	—
% Hepatitis C PCR^††^positive	49.6	—
% Syphilis antibodies	0.7	—

*p value < 0.05; **p value < 0.001.

^†^IVDU: intravenous drug user.

^††^PCR: polymerase chain reaction (test).

**Table 3:** Characteristics of antenatal care.

Characteristics	Result	National data
Mean gestation at first visit, weeks	19.8	—
Mean antenatal visits	6.5	—
% had 5 visits or more	61.2	91.9**
% who had none	7.2	0.3**
% First antenatal visit at 20 weeks or less	56.1	—
% Pregnancy complications	61.9	—
% Hypertension	9.4	—
% Gestational diabetes	2.2	—
% Premature rupture of membranes	11.5	—
% Intrauterine growth restriction	10.1	—
% Antepartum haemorrhage	8.6	—
% Fetal distress	14.4	—
% Prenatal child at risk notifications	29.5	—

*p value < 0.05; **p value < 0.001.

**Table 4:** Characteristics of labour and delivery.

Characteristics	Result	National data
% Booked delivery	93.5	—
% Breech presentation	7.9	4.0*
Labour		
% Spontaneous	61.9	56.6
% Induced	25.9	25.3
% No labour	12.2	18.1
% Caesarean section delivery	28.8	30.9
% Instrumental vaginal delivery	10.8	11.1
% Pain relief for labour	78.4	74.8
Median postnatal length of stay, days	7.0	3.0**

*p value < 0.05; **p value < 0.001.

**Table 5:** Neonate characteristics at birth.

Characteristics	Result	National data
Mean gestational age, weeks	37.6 (range 26–42)	38.8**
% preterm (<37 weeks gestation)	23.7	8.1**
Apgar score at 5 minutes	8.73 (range 3–10)	—
Average birth weight, grams	2798 (range 900–4345)	3374**
Average head circumference, cm	33 (range 24–37.5)	—
Average length, cm	46.9 (range 36–56.5)	—
Birth weight		
% >2500 g	74.8	92.1**
% LBW^†^ (<2500 g)	20.1	6.2**
% Very LBW (<1500 g)	2.2	1.0**
% Extremely LBW (<1000 g)	2.9	0.4**
% Breast milk feeds at birth	45.3	—
% Apgar < 7 at 5 mins after birth	5.8	1.4**
% Admitted to special care nursery/NICU^††^	69.1	14.5**
% NAS^†††^	69.8	—
% Required NAS pharmacotherapy	48.9	—
% Respiratory distress	21.6	—
% Jaundice	20.9	—
% Sepsis	5.8	—
Mean maximum Finnegan's NAS score	7.55 (range 0–18)	—

*p value < 0.05; **p value < 0.001.

^†^LBW: low birth weight.

^††^NICU: neonatal intensive care unit.

^†††^NAS: neonatal abstinence syndrome.

**Table 6:** Hospital discharge and services involved.

Characteristics	Result	National data
Median length of hospital stay, days	9	3
% Length of hospital stay 1–6 days	20.9	93.3**
% Length of hospital stay 7–13 days	46.0	4.2**
% Length of hospital stay 14–20 days	15.8	1.1**
% Length of hospital stay 21–27 days	6.5	0.5**
% Length of hospital stay 28 days or more	8.6	0.8**
Hospital discharge:		
% Home with mother/both parents	77.2	95.0**
% Home in kinship care	2.9	—
% Foster care	10.8	—
% With mother to residential care	2.2	—
% Transferred to another hospital	2.2	3.8
Neonatal death	2.2	2.6^†^
% Child at risk notification post delivery/discharge	38.1	—
% Assumed into care who had sibling/s in care	68.4	—
% Assumed into care with no previous sibling	26.3	—
Services involved at discharge		
Drug and alcohol	77.0	—
Paediatrician	20.9	—
NAS clinic	66.2	—
Child protection	37.4	—
Early childhood	55.4	—
Neonatal early D/C program	18.0	—
Social work	31.7	—
Perinatal and family drug health	12.2	—
Mental health	8.6	—

*p value < 0.05; **p value < 0.001.

^†^NSW comparative data used.
